# Close Relative of Human Middle East Respiratory Syndrome Coronavirus in Bat, South Africa

**DOI:** 10.3201/eid1910.130946

**Published:** 2013-10

**Authors:** Ndapewa Laudika Ithete, Samantha Stoffberg, Victor Max Corman, Veronika M. Cottontail, Leigh Rosanne Richards, M. Corrie Schoeman, Christian Drosten, Jan Felix Drexler, Wolfgang Preiser

**Affiliations:** University of Stellenbosch/National Health Laboratory Service, Tygerberg, South Africa (N.L. Ithete, S. Stoffberg, W. Preiser);; University of Bonn Medical Centre, Bonn, Germany (V.M. Corman, C. Drosten, J.F. Drexler);; University of Ulm, Ulm, Germany (V.M. Cottontail); Durban Natural Science Museum, Durban, South Africa (L.R. Richards);; University of KwaZulu Natal, Durban (M.C. Schoeman)

**Keywords:** Bats, Coronavirus, Middle East respiratory syndrome coronavirus, MERS-CoV, zoonoses, viruses, South Africa

**To the Editor:** The severe acute respiratory syndrome (SARS) outbreak of 2002–03 and the subsequent implication of bats as reservoir hosts of the causative agent, a coronavirus (CoV), prompted numerous studies of bats and the viruses they harbor. A novel clade 2c betacoronavirus, termed Middle East respiratory syndrome (MERS)–CoV, was recently identified as the causative agent of a severe respiratory disease that is mainly affecting humans on the Arabian Peninsula (*1*). Extending on previous work (*2*), we described European *Pipistrellus* bat–derived CoVs that are closely related to MERS-CoV (*3*). We now report the identification of a South Africa bat derived CoV that has an even closer phylogenetic relationship with MERS-CoV.

During 2011–2012, fecal pellets were collected from 62 bats representing 13 different species in the KwaZulu-Natal and Western Cape Provinces of South Africa and stored in RNA*later* solution (Life Technologies, Carlsbad, CA, USA). Details about the bat sample are available in the Technical Appendix. RNA was extracted by using the QIAamp Viral RNA Mini Kit (QIAGEN, Hilden, Germany). Screening for CoVs was done by nested reverse transcription PCR using broadly reactive oligonucleotide primers targeting a conserved region in the *RNA-dependent RNA polymerase* (*RdRp*) gene (online Technical Appendix). PCR results were positive for 5 (8%) of the 62 specimens. PCR amplicons for 4 positive specimens yielded alphacoronavirus sequences related to recently described bat alphacoronaviruses from South Africa (*4*). The other positive specimen, termed PML/2011, was from an adult female *Neoromicia* cf. *zuluensis* bat sampled in 2011; the specimen yielded a novel betacoronavirus (GenBank accession no. KC869678). Technical Appendix Figure 1 shows the distribution of this bat species.

To obtain better phylogenetic resolution, we extended the 398-nt *RdRp* fragment generated by the screening PCR to 816 nt, as described (*5*). PML/2011 differed from MERS-CoV by only 1 aa exchange (0.3%) in the translated 816-nt *RdRp* gene fragment. Thus, PML/2011 was much more related to MERS-CoV than any other known virus. The amino acid sequence of the next closest known relatives of MERS-CoV, from European *Pipistrellus* bats (*3*), differed from MERS-CoV by 1.8%. The amino acid sequences of viruses from *Nycteris* bats in Ghana (*3*) and the 2c prototype bat CoVs, HKU4 and HKU5, from China (*6*) differed by 5.5%–7.7% from MERS-CoV. The smaller 152- to 396-nt *RdRp* fragments of 2c bat CoVs from a *Hypsugo savii* bat in Spain (*7*), bat guano in Thailand (*8*), and a *Nyctinomops* bat in Mexico (*9*) showed no or only partial overlap with the 816-nt fragment generated in this study; thus, a direct comparison could not be done. However, in their respective *RdRp* fragments, these CoVs yielded amino acid sequence distances of 3.5%–8.0% and were thus probably more distant from MERS-CoV than the virus described here. 

A Bayesian phylogenetic analysis of the 816-nt *RdRp* sequence confirmed the close relationship between PML/2011 and MERS-CoV (Figure). Their phylogenetic relatedness was as close as that of SARS-CoV and the most closely related bat coronavirus known, Rs672 from a *Rhinolophus sinicus* bat (Figure). Like PML/2011 and MERS-CoV, Rs672 and SARS-CoV showed only 1 aa exchange in the translated 816-nt *RdRp* fragment. To confirm this relatedness, we amplified and sequenced a short 269-nt sequence encompassing the 3′-terminus of the *spike* gene for PML/2011 (oligonucleotide primers available upon request from the authors). A partial *spike* gene–based phylogeny using this sequence yielded the same topology as that using the partial *RdRp* sequence (Technical Appendix Figure 2). Again, PML/2011 was most closely related to MERS-CoV, showing only a 10.9% aa sequence distance in this gene, which encodes the glycoprotein responsible for CoV attachment and cellular entry. This distance was less than the 13.3% aa sequence distance between MERS-CoV and the European *Pipistrellus* CoVs (*3*) and less than the 20.5%–27.3% aa sequence distance between MERS-CoV and HKU5 and between MERS-CoV and HKU4 (*6*) in the same sequence fragment.

**Figure:**
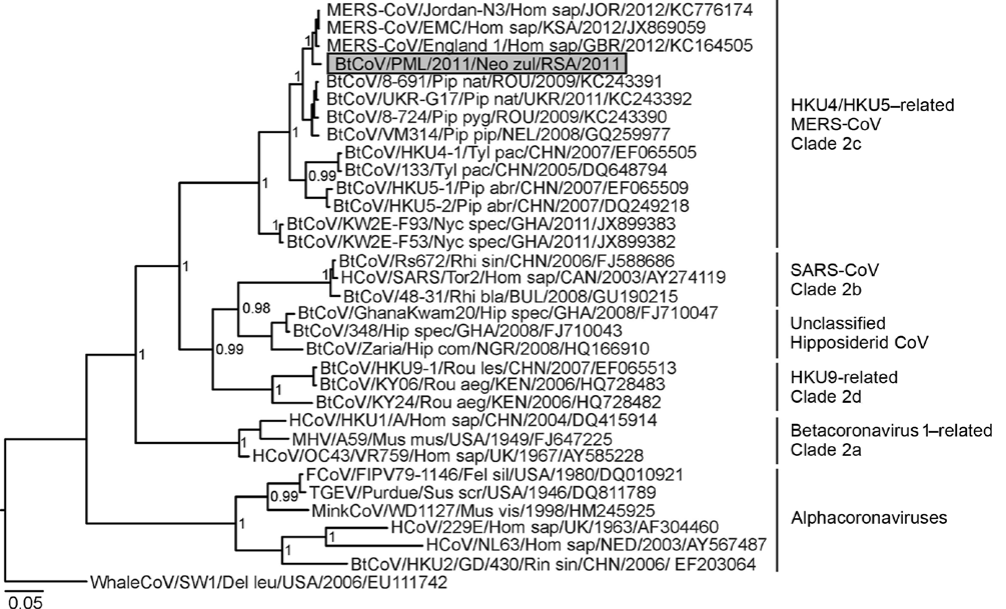
Partial RNA-dependent RNA polymerase (*RdRp*) gene phylogeny, including the novel betacoronavirus from a *Neoromicia zuluensis* bat in South Africa (GenBank accession no. KC869678 for both partial *RdRp* and *spike* gene sequences). The Bayesian phylogeny was done on a translated 816-nt *RdRp* gene sequence fragment, as described (*5*). MrBayes V3.1 (http://mrbayes.sourceforge.net/) was used with a WAG substitution model assumption over 2,000,000 generations sampled every 100 steps, resulting in 20,000 trees, of which 25% were discarded as burn-in. A whale gammacoronavirus was used as an outgroup. The novel *N. zuluensis* bat virus is highlighted in gray. Values at deep nodes represent statistical support from posterior probabilities. Only values >0.9 are shown. Coronavirus clades are depicted to the right of taxa. Scale bar represents genetic distance. MERS-CoV, Middle East respiratory syndrome coronavirus; SARS, severe acute respiratory syndrome; Bt-CoV, bat coronavirus; HCoV, human coronavirus, MHV, mouse hepatitis virus; FCoV, feline coronavirus; TGEV, transmissible gastroenteritis coronavirus.

Our results further support the hypothesis that, like human CoV-229E and SARS-CoV, ancestors of MERS-CoV might exist in Old World insectivorous bats belonging to the family Vespertilionidae, to which the genera *Neoromicia* and *Pipistrellus* belong (*3*). Knowledge of the close relatedness of PML/2011 and MERS-CoV, which contrasts with the more distant relatedness of CoVs in bats from the Americas and Asia, enables speculations of an African origin for bat reservoir hosts of MERS-CoV ancestors. This hypothesis is limited by a global sampling bias, the small sample size, and the single clade 2c betacoronavirus detection in this study. Still, a putative transfer of MERS-CoV ancestors from Africa to the Arabian Peninsula would parallel the transfer of other viruses (e.g., the exportation of Rift Valley fever virus from East Africa, which led to a severe outbreak in Saudi Arabia in 2000) (*10*).

Studies of Vespertilionidae bats and potential intermediate hosts (e.g., carnivores and ungulates, such as camels) are urgently needed to elucidate the emergence of MERS-CoV. Such studies should focus on the Arabian Peninsula and Africa.

Technical Appendix Description of bat sampling, screened bat species, distribution of *Neoromicia zuluensis* bats, and *spike* gene phylogeny of the 2c betacoronavirus clade.

## References

[1] Zaki AM, van Boheemen S, Bestebroer TM, Osterhaus AD, Fouchier RA. Isolation of a novel coronavirus from a man with pneumonia in Saudi Arabia. N Engl J Med. 2012;367:1814–20. 10.1056/NEJMoa1211721 23075143

[2] Reusken CB, Lina PH, Pielaat A, de Vries A, Dam-Deisz C, Adema J, Circulation of group 2 coronaviruses in a bat species common to urban areas in Western Europe. Vector Borne Zoonotic Dis. 2010;10:785–91. 10.1089/vbz.2009.0173 20055576

[3] Annan A, Baldwin HJ, Corman VM, Klose SM, Owusu M, Nkrumah EE. Human betacoronavirus 2c EMC/2012–related viruses in bats, Ghana and Europe. Emerg Infect Dis. 2013;19:456–9. 10.3201/eid1903.121503 23622767 PMC3647674

[4] Geldenhuys M, Weyer J, Nel LH, Markotter W. Coronaviruses in South African bats. Vector Borne Zoonotic Dis. 2013;13:516–9. 10.1089/vbz.2012.1101 23473214

[5] Drexler JF, Gloza-Rausch F, Glende J, Corman VM, Muth D, Goettsche M, Genomic characterization of severe acute respiratory syndrome–related coronavirus in European bats and classification of coronaviruses based on partial RNA-dependent RNA polymerase gene sequences. J Virol. 2010;84:11336–49. 10.1128/JVI.00650-10 20686038 PMC2953168

[6] Woo PC, Wang M, Lau SK, Xu H, Poon RW, Guo R, Comparative analysis of twelve genomes of three novel group 2c and group 2d coronaviruses reveals unique group and subgroup features. J Virol. 2007;81:1574–85. 10.1128/JVI.02182-06 17121802 PMC1797546

[7] Falćon A, Vazquez-Moron S, Casas I, Aznar C, Ruiz G, Pozo F, Detection of alpha and betacoronaviruses in multiple Iberian bat species. Arch Virol. 2011;156:1883–90. 10.1007/s00705-011-1057-1 21766197 PMC3181409

[8] Wacharapluesadee S, Sintunawa C, Kaewpom T, Khongnomnan K, Olival KJ, Epstein JH. Group C betacoronavirus in bat guano fertilizer, Thailand. Emerg Infect Dis. 2013; Epub ahead of print. 10.3201/eid1908.130119 PMC3739538 23880503

[9] Anthony SJ, Ojeda-Flores R, Rico-Chavez O, Navarrete-Macias I, Zambrana-Torrelio C, Rostal MK, Coronaviruses in bats from Mexico. J Gen Virol. 2013;94:1028–38. 10.1099/vir.0.049759-0 23364191 PMC3709589

[10] Bird BH, Khristova ML, Rollin PE, Ksiazek TG, Nichol ST. Complete genome analysis of 33 ecologically and biologically diverse Rift Valley fever virus strains reveals widespread virus movement and low genetic diversity due to recent common ancestry. J Virol. 2007;81:2805–16. 10.1128/JVI.02095-06 17192303 PMC1865992

