# Supplementary material for: Close Relative of Human Middle East Respiratory Syndrome Coronavirus in Bat, South Africa
Source: Emerg Infect Dis. 2013 Oct;19(10):1697–9. doi: 10.3201/eid1910.130946 (PMC3810765; doi:10.3201/eid1910.130946)
Supplement: Technical Appendix — Description of bat sampling, screened bat species, distribution of Neoromicia zuluensis bats, and spike gene phylogeny of the 2c betacoronavirus clade. [file 13-0946-Techapp-s1.pdf]

Publisher: CDC; Journal: Emerging Infectious Diseases
Article Type: Letter; Volume: 19; Issue: 10; Year: 2013; Article ID: 13-0946
DOI: 10.3201/eid1910.130946; TOC Head: Letters to the Editor
Article DOI: http://dx.doi.org/10.3201/eid1910.130946

Close Relative of Human Middle East Respiratory Syndrome Coronavirus in Bat, South Africa

Technical Appendix

Sampling

Bats were sampled between November 2010 and mid-2012 at caves in Table Mountain National Park and in Millwood forest, Garden Route National Park (permit no. 11LB_SEI01), at Phinda Private Game Reserve in KwaZulu Natal (permit no. OP2021/2011) and in Greyton, Western Cape (permit no. AAA007-00373-0035).

Bats were captured during emergence from roof roosts or cave entrances using a harp trap, hand-net or mist nets. Animal handling and sample collection was done in accordance with accepted international guidelines for mammals as set out in Sikes et al. (*1*).

Individuals were then placed in individual cloth bags for up to 3 hours to collect faecal pellets. Faecal pellets were removed from each bag using sterile forceps and suspended in 1.0ml of RNAlater in a 2ml cryovial before transport to the laboratory in Tygerberg and virological testing under institutional clearance (ref. SU-ACUM12-00001).

In the Western Cape, all bats were released unharmed. In Phinda, they were euthanized with halothane and retained as accessioned voucher specimens in the mammal collection of the Durban Natural Science Museum. Associated specimen derivatives (e.g. faecal pellets) were obtained through a museum loan (loan no. M201011_1).

Species were determined based on morphological features following current systematics (*2*). Forearm length and body mass were measured and the age (adult or juvenile) was determined by assessing the presence of cartilaginous epiphyseal plates in the finger bones (*3*). The sex and reproductive status of each individual was also recorded.

Publisher: CDC; Journal: Emerging Infectious Diseases
Article Type: Letter; Volume: 19; Issue: 10; Year: 2013; Article ID: 13-0946
DOI: 10.3201/eid1910.130946; TOC Head: Letters to the Editor

Initial nested RT-PCR screening used broadly reactive oligonucleotide primers targeting the coronavirus RNA-dependent RNA polymerase gene (*4*).

References

1. Sikes RS, Gannon WL; Animal Care and Use Committee of the Amercian Society of Mammalogists. Guidelines of the American Society of Mammalogists for the use of wild mammals in research. Journal of Mammalogy. 2001;92:235–53.

2. Roehrs ZP, Lack JB, Van Den Bussche RA. Tribal phylogenetic relationships within Vespertilioninae (Chiroptera: Vespertilionidae) based on mitochondrial and nuclear sequence data. Journal of Mammalogy. 2010;91:1073–92. http://dx.doi.org/10.1644/09-MAMM-A-325.1

3. Brunet-Wilkinson AK, Wilkinson GS. Methods for age estimation and the study of senescence in bats. In: Kunz TH, Parsons S, editors. Ecological and behavioral methods for the study of bats, second edition. Chap. 15. Baltimore: The John Hopkins University Press; 2009.

4. de Souza Luna LK, Heiser V, Regamey N, Panning M, Drexler JF, Mulangu S, et al. Generic detection of coronaviruses and differentiation at the prototype strain level by reverse transcription–PCR and nonfluorescent low-density microarray. J Clin Microbiol. 2007;45:1049–52.

Technical Appendix Table. South African bat species screened

Family	Species	Number of specimens
Insectivorous bats		
Molossidae	*Chaerephon pumilus*	5
	Mops condylurus	1
	Tadarida aegyptiaca	3
Hipposideridae	*Hipposideros caffer*	4
Miniopteridae	*Miniopterus natalensis*	13
Nycteridae	*Nycteris thebaica*	1
Rhinolophidae	*Rhinolophus clivosus*	2
	Rhinolophus darlingi	2
Vespertilionidae	*Neoromicia capensis*	10
	Neoromicia nana	6
	Neoromicia cf. *zuluensis*	1
	Scotophilus viridis	3
Fruit bats		
Pteropodidae	*Rousettus aegyptiacus*	12
Total		62

Publisher: CDC; Journal: Emerging Infectious Diseases
Article Type: Letter; Volume: 19; Issue: 10; Year: 2013; Article ID: 13-0946
DOI: 10.3201/eid1910.130946; TOC Head: Letters to the Editor

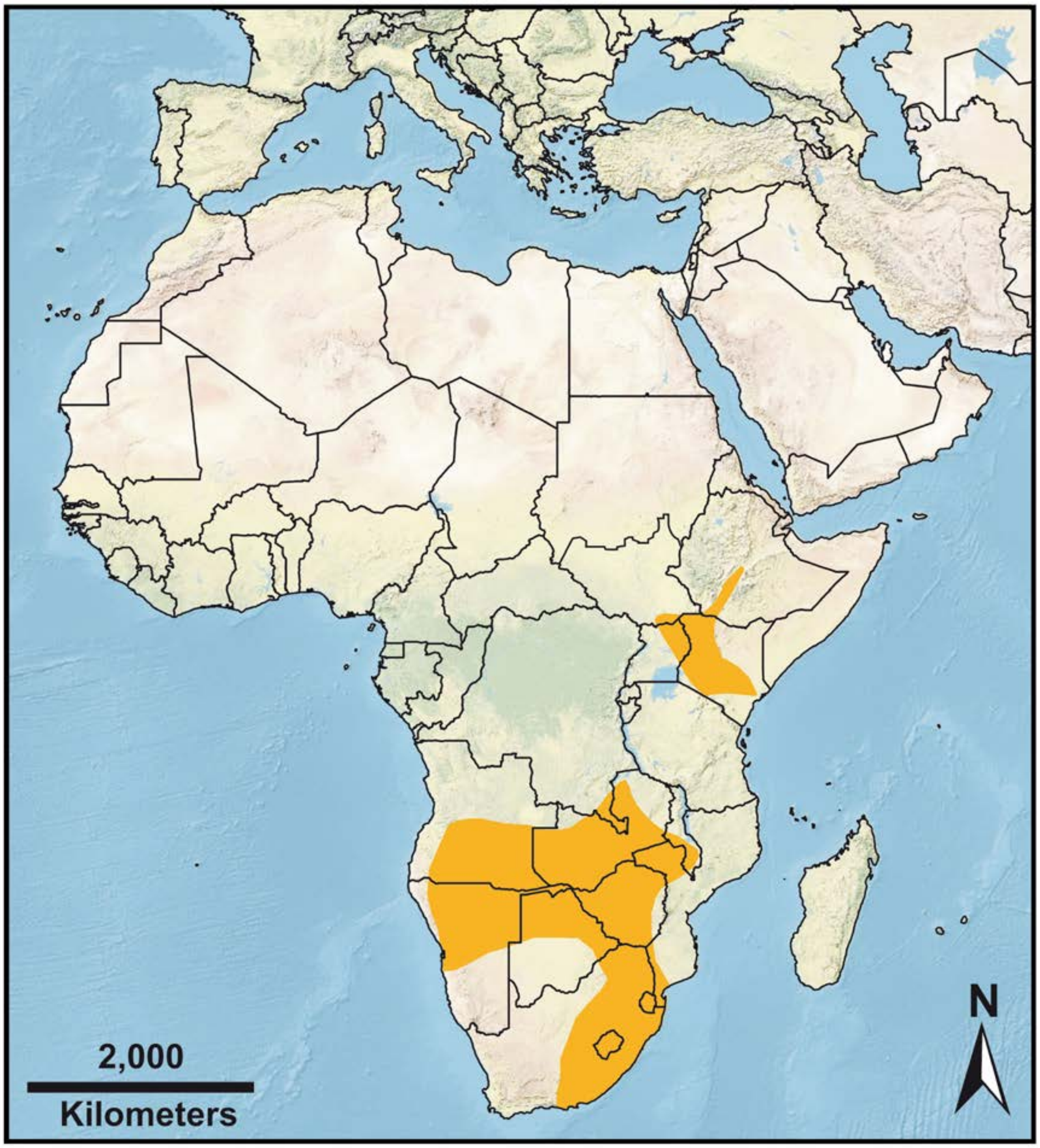

Technical Appendix Figure 1. Distribution map of *Neoromicia zuluensis*, Africa. Adapted from the IUCN Red List of Threatened Species. Version 2012.2 (www.iucnredlist.org) using ArcGIS software by Esri.

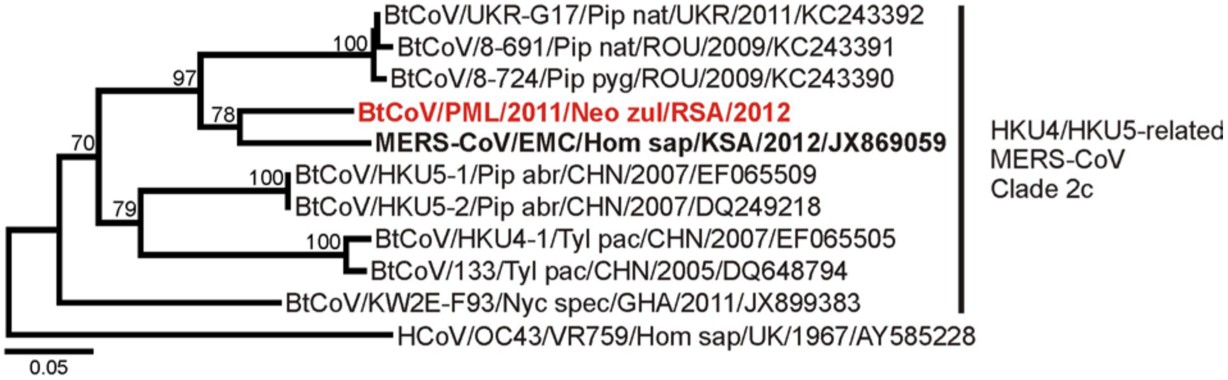

Technical Appendix Figure 2. Partial *Spike* gene phylogeny of the 2c betacoronavirus clade including the novel bat virus. The neighbor-joining phylogeny of the partial *Spike* gene of clade 2c coronaviruses including the novel South African *Neoromicia* coronavirus was done using a nucleotide percentage distance substitution model and the complete deletion option in MEGA5. The novel bat virus is shown in bold and red, Middle East respiratory syndrome (MERS)–CoV in bold only. Scale bar represents percentage nucleotide distance. The analysis comprised a total of 138 nucleotides corresponding to positions 25,379-25,517 in MERS-CoV. HCoV-OC43 was used as an outgroup. Oligonucleotide sequences of primers used for amplification of partial *Spike* gene sequences are available upon request. Of the larger 269 nucleotide fragment which was amplified, only the 3′-terminus of the *Spike* gene was included into the phylogenetic analysis to avoid a bias from potential recombination known to occur frequently at the borders of the *Spike* gene. Values at deep nodes represent statistical support of grouping by percentage of 1,000 bootstrap replicates.
